# Quaint Cures and Prescriptions

**Published:** 1908-11-28

**Authors:** 


### Medical Antiquities.

### QUAINT CURES AND PRESCRIPTIONS.

Every time seems to have had its Lourdes and every age its quackeries. We find, for example, during the classic era in Greece, that at Epidaurus many wonderful cures were effected in the temple. A man with one eye has a vision, and departs seeing with both eyes. A boy is cured of stone by being licked by the temple dogs, while the lick of a snake heals another man's toe. Gout is cured by leeches, administered in the patient's drink by his stepmother.

Perhaps the quaintest of all prescriptions are those related by Sir Thomas Browne. This remarkable writer, whose conception of man as "a noble animal, splendid in ashes and pompous in the grave," is as flattering as it is sublime, dearly loved whatsoever was fantastic, superstitious, or bizarre, and seems to have taken endless trouble to collect the most weird and complicated treatments of his own and former times. Pierius, he tells us, gave as an antidote against the sting of a scorpion, that a man should sit upon an ass with his face to the tail, thus causing the pain to leave the man and pass into the beast. Sammonicus prescribes an "uncomfortable receipt" for a quartan ague: to lay the fourth book of the Iliad of Homer under the sufferer's head.

The collyrium of Albertus promised the power of sight in the dark. This was effected "by the right eye of a hedgehog, boiled in oil, and preserved in a brazen vessel." The precept of Kiranides is most remarkable. The left stone of a weasel, wrapt up in the skin of a she mule "is able to secure incontinency from conception."

It is but just to Sir Thomas Browne to say that he disclaims any personal belief in these ingenious suggestions. But in all his writings he dwells very lovingly, and in a spirit of grave philosophical inquiry, upon every manner of superstition and legendary humbug. He is obviously impressed by the advice given to Democritus by an oracle, that for the falling sickness, he commended "the maggot in a goat's head." Nor is he much opposed to a variety of prescriptions for the cure of blindness. Aper was counselled to make a collyrium with the blood of a white cock and honey and to apply it to his eyes for three days. "Julian was cured of blood-spitting by honey and nuts taken from the altar, while Lucius, for a pain in his side, applied ashes from the altar with wine."

The "sympathetic powder," which became celebrated about the middle of the seventeenth century, affords a most instructive example of the hold which witchcraft and superstition still held upon the medical mind. The wound, for example, was simply closed and the powder put upon the sword that caused it. This treatment was much followed until some daring innovator closed the wound and omitted to powder the sword.

A very simple cure for warts was "to rub the hands before the moon, which proves," says Sir Kenelme Digby, "that the rays of the moon are cold and moist." "To prevent nightmare, we hang up a hollow stone in our stables, while for amulets against agues, we use the chips of gallows and places of execution." Even Francis Bacon related a story about the charming away of his warts. The Hon. Robert Boyle was pleased to attribute the cure of a hæmorrhage to wearing "some moss from a dead man's skull."

Much of ancient lore and curious beliefs is to be found in the pages of Sir Thomas Browne, and few indeed are the authors whose style is at once so rhythmical and sonorous. He possessed in no small degree that curiosity "to find out things," which is indispensable to the true scientist, but at the same time he spared no pains to render the form in which he expressed his thoughts pleasing and artistic.

